# Tetraethylammonium-Sensitive ventricular repolarization in diabetic rat myocardium: effects of rosiglitazone

**DOI:** 10.20945/2359-4292-2026-0120

**Published:** 2026-10-11

**Authors:** Servet Kavak, Mehmet Ali Ijik, Mustafa Emre

**Affiliations:** 1 Department of Biophysics, Faculty of Medicine, Izmir Bakircay University, Izmir, Türkiye; 2 Department of Cardiology, Mardin State Hospital, Mardin, Türkiye; 3 Department of Biophysics, Faculty of Medicine, Çukurova University, Adana, Türkiye

**Keywords:** Diabetes mellitus, diabetic cardiomyopathy, ventricular repolarization, potassium channels, tetraethylammonium, rosiglitazone, cardiac electrophysiology

## Abstract

**Objective:**

To examine ventricular action-potential responses to tetraethylammonium (TEA), a non-selective potassium-channel blocker, in streptozotocin-induced diabetic rats and determine whether rosiglitazone modifies these responses.

**Materials and methods:**

Male Wistar rats were assigned to Control (C), Control plus Rosiglitazone (C+RSG), Diabetic (D), or Diabetic plus Rosiglitazone (D+RSG) groups. Diabetes was induced with streptozotocin (45 mg/kg, intravenous). Resting membrane potential (RMP), action potential amplitude (APA), and half-repolarization time (½RT) were recorded from isolated left ventricular papillary muscles before and after TEA administration (2 mmol/L).

**Results:**

At week 8, blood glucose was 563 ± 29 mg/dL in diabetic rats and 98 ± 6 mg/dL in controls (p < 0.001). Rosiglitazone reduced the diabetic value to 336 ± 25 mg/dL (*p* < 0.05 vs. D). TEA increased ½RT from 18 ± 1 to 38 ± 2 ms in controls (+20 ms) and from 60 ± 3 to 65 ± 3 ms in diabetic myocardium (+5 ms). Diabetic preparations had higher post-TEA ½RT because repolarization was prolonged at baseline; however, the absolute TEA-associated increment was smaller than in controls. TEA also increased APA in untreated control and diabetic preparations.

**Conclusion:**

Diabetes was associated with marked baseline prolongation of ventricular repolarization. The smaller absolute ½RT increment in diabetic myocardium does not support enhanced TEA responsiveness. Because TEA is non-selective, the findings reflect an integrated response to TEA-sensitive potassium conductances rather than dysfunction of a specific current subtype. Rosiglitazone modified selected electrophysiological responses without normalizing diabetic repolarization.

## INTRODUCTION

Diabetes mellitus (DM) is a significant global health challenge associated with a substantial increase in cardiovascular morbidity and mortality. In addition to accelerating atherosclerosis and heart failure, diabetes promotes the development of diabetic cardiomyopathy, a distinct myocardial disorder characterized by metabolic abnormalities, oxidative stress, mitochondrial dysfunction, inflammation, fibrosis, and electrical remodeling (^1^,^2^,^3^,^4^,^5^).

Accumulating evidence shows that diabetic cardio-myopathy is accompanied by pronounced alterations in cardiac electrophysiology, leading to increased susceptibility to ventricular arrhythmias and sudden cardiac death (^6^,^7^,^8^,^9^). Clinical studies have reported increased QT interval duration, QT dispersion, and ventricular ectopic activity in diabetic patients, suggesting impaired repolarization reserve and increased electrical instability (^10^,^11^).

Cardiac electrical activity depends on the coordinated activation of multiple ion channels that regulate membrane excitability and action potential morphology. Ventricular repolarization is primarily governed by potassium currents, including the transient outward potassium current (Ito), rapid delayed rectifier potassium current (IKr), slow delayed rectifier potassium current (IKs), and inward rectifier potassium current (IK1) (^7^,^8^,^12^). These currents collectively determine action potential duration, membrane stability, and repolarization reserve, protecting the myocardium against arrhythmogenesis.

Diabetes-induced electrophysiological remodeling has been linked to changes in potassium channel expression and function. Experimental studies have shown reductions in Ito density and dysregulation of Kv4.2, Kv4.3, Kv1.5, and Kir2.x channel subunits in diabetic myocardium (^6^,^13^,^14^). Chronic hyperglycemia, advanced glycation end-products, oxidative stress, mitochondrial dysfunction, and inflammatory signaling further contribute to ion channel remodeling and impaired electrical stability (^1^,^2^,^3^,^14^,^15^).

Mitochondrial dysfunction has emerged as a central mechanism linking metabolic abnormalities to electrical remodeling in diabetic hearts. Increased production of reactive oxygen species, altered calcium handling, impaired ATP generation, and disrupted redox signaling pathways may directly affect potassium channel activity and ventricular repolarization (^13^,^16^,^17^,^18^).

Tetraethylammonium chloride (TEA) is a classical but concentration-dependent and relatively non-selective potassium-channel blocker used to probe the aggregate contribution of TEA-sensitive outward conductances to membrane repolarization (^8^,^19^). At 2 mmol/L, TEA may affect more than one potassium-current component and cannot identify an individual current or channel subtype. Accordingly, TEA-induced changes in action-potential morphology are interpreted here as an integrated functional response to broad potassium-conductance inhibition, not as direct evidence of remodeling of a specific potassium channel. The baseline prolongation of repolarization in diabetic tissue is compatible with reduced repolarization capacity; however, the incremental TEA response must be evaluated separately from the final post-TEA value.

Rosiglitazone is a peroxisome proliferator-activated receptor gamma (PPARγ) agonist widely used to improve insulin sensitivity and glycemic control. Beyond its metabolic effects, accumulating evidence suggests that PPARγ signaling modulates oxidative stress pathways, mitochondrial bioenergetics, calcium handling, inflammatory responses, and ion channel expression (^13^,^20^,^21^,^22^). Several studies have reported direct electrophysiological effects of rosiglitazone on cardiac tissue, including modulation of potassium channel activity and ventricular action potential characteristics. In a previous study, rosiglitazone partially reversed diabetes-induced abnormalities in ventricular papillary muscle electrophysiology and improved action potential properties in streptozotocin (STZ)-induced diabetic rats, suggesting that PPARγ activation may affect myocardial electrical activity independently of glycemic control (^23^). These findings support further investigation into the interaction between rosiglitazone treatment and potassium channel-dependent ventricular repolarization mechanisms.

Although diabetes-associated ventricular electrical remodeling has been extensively studied, information regarding the interaction between TEA-sensitive potassium conductances and rosiglitazone-mediated electrophysiological modulation in ventricular myocardium remains limited. Clarifying these mechanisms could enhance the understanding of diabetes-associated impairment of repolarization reserve and the increased arrhythmogenic susceptibility observed in diabetic cardiomyopathy.

Therefore, the present study aimed to evaluate the effects of non-selective TEA-sensitive potassium-conductance blockade on ventricular action potential characteristics in isolated papillary muscles from streptozotocin-induced diabetic rats and to determine whether rosiglitazone modifies the integrated electrophysiological response under diabetic conditions.

## MATERIALS AND METHODS

### Study design

This experimental animal study was conducted to investigate the contribution of TEA-sensitive potassium conductances to ventricular repolarization in diabetic myocardium and to evaluate the modulatory effects of rosiglitazone on potassium channel-dependent electrophysiological responses. The study was conducted and reported according to the ARRIVE 2.0 (Animal Research: Reporting of In Vivo Experiments) guidelines (^24^).

### Animals

Thirty-two adult male Wistar albino rats weighing 250-320 g were housed under standardized laboratory conditions (22 ± 2 °C, relative humidity 55 ± 10%, 12-h light/dark cycle) with standard feed and tap water *ad libitum.*

### Ethical approval

All experimental procedures were conducted in accordance with the National Institutes of Health Guide for the Care and Use of Laboratory Animals (^25^) and complied with internationally accepted principles of animal welfare. The protocol was approved by the Ethics Committee of the Çukurova University Medical Sciences Experimental Research and Application Center in Adana, Turkey (approval no. 163/2005). All efforts were made to minimize animal suffering and to reduce the number of animals used.

### Experimental induction of diabetes

Experimental DM was induced by a single intravenous injection of STZ (45 mg/kg; Sigma Chemical Co., USA) freshly dissolved in 0.01 M sodium citrate buffer (pH 4.5). One week after STZ administration, fasting blood glucose concentrations were measured using a portable glucometer (Accu-Chek^®^, Roche Diagnostics, Germany). Animals exhibiting fasting blood glucose levels >200 mg/dL on three consecutive measurements were considered diabetic and included in the study.

### Experimental groups, randomization, and allocation

After confirmation of diabetes, animals were randomly assigned using a computer-generated schedule to one of four experimental groups: control (C), Control + rosiglitazone (C+RSG), diabetic (D), or diabetic + rosiglitazone (D+RSG). Each group contained eight animals. Randomization was performed by an investigator not involved in subsequent data collection, elec-trophysiological recordings, or statistical analyses to minimize allocation bias.

### Rosiglitazone treatment

Rosiglitazone (GlaxoSmithKline, UK) was administered orally by gavage (4 mg/kg) twice daily for eight weeks. Body weight and fasting blood glucose concentrations were recorded weekly throughout the study.

### Sample size determination

Sample size was determined using a priori power analysis performed with G*power software (v. 3.1) (^26^). Based on previous studies evaluating ventricular repolarization parameters in diabetic myocardium, a minimum sample size of seven animals per group was required to achieve a statistical power of 80% at an alpha level of 0.05. Thus, eight animals were included in each experimental group to account for potential losses.

### Papillary muscle preparation

After the eight-week treatment period, animals were deeply anesthetized and euthanized by cervical dislocation. Hearts were rapidly excised and placed in ice-cold, oxygenated Krebs-Henseleit solution. Left ventricular papillary muscles were carefully dissected and mounted in a temperature-controlled Plexiglas chamber for continuous superfusion. Preparations were su-perfused at 6-8 mL/min with Krebs-Henseleit solution containing (mmol/L): NaCl 113, KCl 4.7, MgSO4 · 7H2O 1.2, CaCl2 · 2H2O 1.9, KH2PO4 1.2, NaHCO3 25, and glucose 11.5. The solution was continuously bubbled with 95% O2 and 5% CO2 to maintain a physiological pH of 7.4. Bath temperature was maintained at 30 ± 0.4 °C throughout all experiments.

### Intracellular electrophysiological recordings

Transmembrane action potentials were recorded using conventional intracellular glass microelectrodes filled with 3 M KCl (resistance 5-15 MΩ). Papillary muscles were stimulated with rectangular electrical pulses (1 ms duration) at twice the diastolic threshold and a constant frequency of 1 Hz. Signals were amplified via a high-input impedance amplifier (Nihon Kohden, Japan) equipped with capacitance compensation, displayed on an oscilloscope, digitized using an analog-to-digital converter, and analyzed with a computer-assisted data acquisition system. Only recordings with stable resting membrane potentials (RMP) and consistent action potential morphology were included in the analysis.

### Electrophysiological parameters

The evaluated parameters included RMP (mV), action potential amplitude (APA, mV), and half-repolarization time (·RT, ms). The RMP values were used to assess myocardial membrane stability, APA reflected cellular excitability and depolarization, and ½RT represented ventricular repolarization dynamics and served as the primary outcome for evaluating the effects of TEA-sensitive potassium channel blockade under both normal and diabetic conditions. Baseline measurements were obtained prior to pharmacological intervention **(Figure 1).**

### Tetraethylammonium administration

Tetraethylammonium chloride (TEA-Cl) was freshly prepared on the day of the experiment and added to the superfusion solution at a final concentration of 2 mmol/L. This concentration was selected to inhibit a broad set of TEA-sensitive potassium conductances involved in ventricular repolarization (^19^). Because TEA at this concentration is not subtype-selective, the experiment was designed as a functional perturbation of aggregate TEA-sensitive potassium conductance; it cannot distinguish Ito, IKr, IKs, IK1, or individual Kv/Kir channel contributions.

### Outcome measures

The primary outcome was ½RT. Secondary outcomes included RMP and APA. Weekly body weight and fasting blood glucose concentrations were also monitored during the experimental period.

Electrophysiological recordings, data acquisition, and statistical analyses were performed by investigators blinded to group allocation. Treatment codes were revealed only after all statistical analyses were complete.

### Statistical analysis

Data are expressed as mean ± standard error of the mean (SEM). Normality of model residuals was assessed using the Shapiro-Wilk test. For each electrophysiological endpoint (RMP, APA, and ½RT), the effects of experimental group (C, C+RSG, D, and D+RSG; between-subject factor), TEA condition (before vs. after TEA; within-subject factor), and the group × TEA interaction were evaluated using a two-way mixed-design repeated-measures ANOVA. When a significant omnibus effect was detected, multiplicity-adjusted post hoc comparisons were performed using Sidak's procedure. Effect estimates are reported with 95% confidence intervals where applicable. Weekly body weight and glucose data were analyzed separately using an appropriate group × time repeated-measures model. Statistical analyses were performed using GraphPad Prism version 9.0 (GraphPad Software Inc., San Diego, CA, USA), and two-tailed p < 0.05 was considered statistically significant.

**Figure 1:**
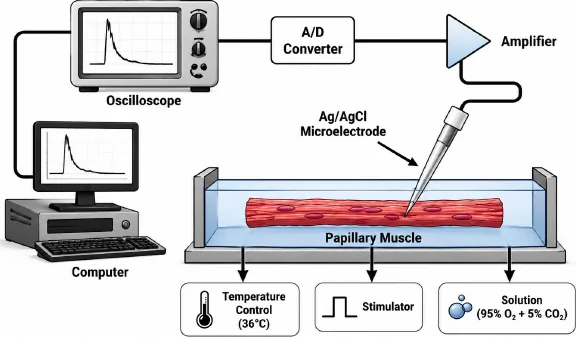
**Experimental setup for intracellular electrophysiological recording.** Schematic illustration of the experimental setup for intracellular recordings in isolated rat ventricular papillary muscles. Preparations were continuously superfused with oxygenated Krebs-Henseleit solution and electrically stimulated throughout the experiment. Transmembrane action potentials were digitized and analyzed using a computer-based acquisition system.

A/D: analog-to-digital.

### ARRIVE 2.0 Compliance statement

No animals were excluded from the final statistical analyses, and all randomized animals completed the protocol. No adverse events occurred during the study. Experimental endpoints and statistical analyses were predefined before data collection in line with ARRIVE 2.0 recommendations (^24^).

## RESULTS

## Body weight changes

Weekly body weight changes are presented in **Figure 2.** At baseline, there were no significant differences in body weight among the experimental groups (*p* > 0.05). Throughout the eight-week experimental period, control animals displayed progressive physiological weight gain, with the greatest increase observed in the rosiglitazone-treated control group, which achieved approximately 131% of baseline body weight by week 8. In contrast, STZ-induced diabetic rats exhibited significantly impaired body weight gain. Body weight progressively declined after diabetes induction, reaching approximately 88% of baseline values by the end of the study. Rosiglitazone treatment partially mitigated diabetes-associated weight loss. Diabetic rats receiving rosiglitazone consistently maintained significantly greater body weights than untreated diabetic animals throughout the experimental period *(p* < 0.05), although their values remained lower than those of non-diabetic controls.

**Figure 2:**
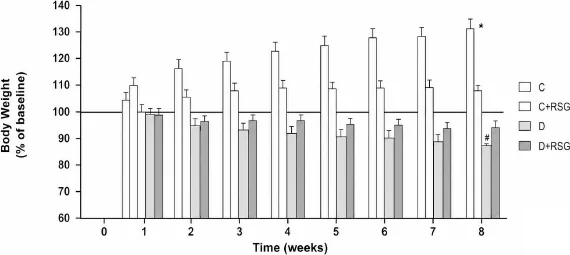
**Effects of rosiglitazone on body weight in streptozotocin-induced diabetic rats.** Weekly body weight is expressed as a percentage of baseline, over the 8-week experimental period. Control animals showed progressive weight gain, while diabetic rats exhibited impaired weight gain and progressive weight loss. Rosiglitazone treatment partially attenuated diabetes-associated weight reduction. Data are presented as mean ± standard error of the mean.

C: control; C+RSG: control plus rosiglitazone; D: diabetic; D+RSG: diabetic plus rosiglitazone.

*p < 0.05 vs. control group; ^#^p < 0.05 vs. diabetic group.

## Blood glucose levels

Changes in fasting blood glucose concentrations during the experimental period are shown in **Figure 3.** Before STZ administration, fasting blood glucose concentrations were comparable in all groups (*p* > 0.05). One week after STZ injection, diabetic animals developed marked hyperglycemia, confirming successful induction of DM. Blood glucose concentrations progressively increased in untreated diabetic rats and remained significantly elevated throughout the eight-week study, with values exceeding 550 mg/dL (*p* < 0.05 vs. control). Rosiglitazone treatment significantly reduced fasting blood glucose concentrations in diabetic animals compared with untreated diabetic rats *(p* < 0.05). Nevertheless, glucose concentrations in treated diabetic animals remained substantially higher than in non-diabetic controls. No significant changes in blood glucose concentrations were observed in non-diabetic animals receiving rosiglitazone.

**Figure 3:**
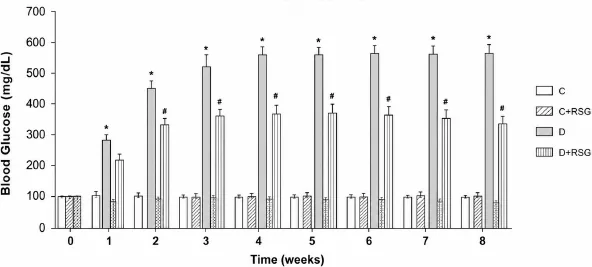
**Blood glucose levels during the experimental period.** Weekly fasting blood glucose concentrations measured over the 8-week experimental period. Streptozotocin caused persistent hyperglycemia in diabetic animals. Rosiglitazone significantly reduced blood glucose concentrations but did not normalize glycemic status. Data are presented as mean ± standard error of the mean.

C: control; C+RSG: control plus rosiglitazone; D: diabetic; D+RSG: diabetic plus rosiglitazone.

*p < 0.05 vs. control group; ^#^*p <* 0.05 vs. diabetic group.

## Effects of TEA on resting membrane potential

Under baseline conditions, mean RMP was -68 mV in control preparations and -71 mV in diabetic preparations. The direction and statistical significance of the group effect should be interpreted from the revised group × TEA model rather than described qualitatively as depolarization. Following TEA exposure, RMP changed from -68 to -63 mV in C, -66 to -61 mV in C+RSG, -71 to -61 mV in D, and -63 to -59 mV in D+RSG **(Figure 4).** These within-group changes are presented descriptively; differences in TEA response among groups require support from the group × TEA interaction and multiplicity-adjusted post hoc comparisons. No ranking of the RMP changes as "largest", "modest", or "similar" is made without a direct statistical comparison of the change estimates.

**Figure 4:**
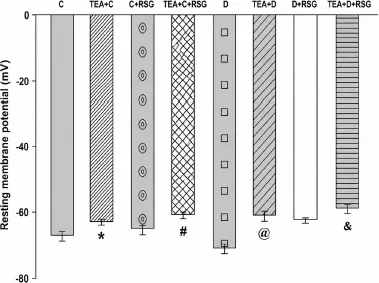
**Effects of tetraethylammonium and rosiglitazone on resting membrane potential.** Resting membrane potential recorded from isolated ventricular papillary muscles from control and diabetic rats before and after tetraethylammonium administration. Diabetes caused membrane depolarization, while rosiglitazone partially restored resting membrane potential toward control values. Tetraethylammonium altered membrane polarization in both control and diabetic myocardium. Data are presented as mean ± standard error of the mean.

C: control; RSG: rosiglitazone; D: diabetic; TEA: tetraethylammonium.

*p < 0.05 vs. control group; ^#^*p <* 0.05 vs. control plus rosiglitazone group; ^@^p *<* 0.05 vs. diabetic group; ^&^*p <* 0.05 vs. diabetic plus rosiglitazone group.

## Effects of TEA on action potential amplitude

Baseline mean APA values were 68 mV in C, 79 mV in C+RSG, 68 mV in D, and 78 mV in D+RSG. Group differences are interpreted according to the revised omnibus model and adjusted post hoc comparisons. Following TEA administration, mean APA changed from 68 to 72 mV in C and from 68 to 74 mV in D. Within-group statistical findings should be reported using multiplicity-adjusted contrasts derived from the revised group × TEA model. Mean APA changed from 79 to 78 mV in C+RSG and from 78 to 80 mV in D+RSG **(Figure 5).** Qualitative labels such as "modest" or "similar" have been removed because they require direct statistical comparison.

**Figure 5:**
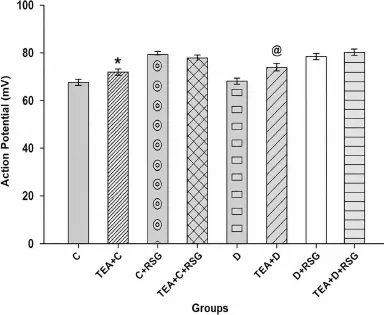
Effects of tetraethylammonium and rosiglitazone on action potential amplitude. Action potential amplitude recorded from isolated ventricular papillary muscles from control and diabetic rats before and after tetraethylammonium administration. Diabetes reduced myocardial excitability, while rosiglitazone partially improved action potential amplitude. Tetraethylammonium altered electrophysiological responses, highlighting the contribution of potassium conductances to action potential generation. Data are presented as mean ± standard error of the mean.

C: control; RSG: rosiglitazone; D: diabetic; TEA: tetraethylammonium.

*p < 0.05 vs. control group; ^@^*p <* 0.05 vs. diabetic group.

## Effects of TEA on half-repolarization time

At baseline, mean ½RT was 18 ms in control myocardium and 60 ms in diabetic myocardium, indicating substantially longer baseline repolarization in the diabetic group (adjusted between-group p value to be reported from the revised model). After TEA exposure, mean ½RT changed from 18 to 38 ms in C (+20 ms), from 29 to 46 ms in C+RSG (+17 ms), from 60 to 65 ms in D (+5 ms), and from 55 to 69 ms in D+RSG (+14 ms) **(Figure 6).** Significance of the within-group contrasts and differences among these changes should be based on the group × TEA interaction and Sidak-adjusted comparisons.

**Figure 6:**
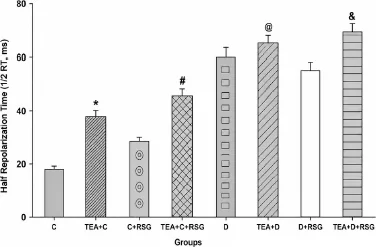
**Effect of tetraethylammonium on half-repolarization time.** Half-repolarization time (½RT) in isolated ventricular papillary muscles before and after tetraethylammonium (TEA; 2 mmol/L). Mean ½RT changed from 18 to 38 ms in C (+20 ms), 29 to 46 ms in C+RSG (+17 ms), 60 to 65 ms in D (+5 ms), and 55 to 69 ms in D+RSG (+14 ms). Diabetic myocardium had longer baseline and post-TEA values, but the absolute increment in untreated diabetes was smaller than in untreated controls. TEA is non-selective; the figure does not identify a specific current or channel subtype. Inferential comparisons must be based on the revised group × TEA repeated-measures model.

C: control; C+RSG: control plus rosiglitazone; D: diabetic; D+RSG: diabetic plus rosiglitazone; TEA: tetraethylammonium; ½RT: half-repolarization time.

*p < 0.05 vs. control baseline; ^#^*p <* 0.05 vs. control plus rosiglitazone baseline; ^@^*p <* 0.05 vs. diabetic baseline; ^&^*p <* 0.05 vs. diabetic plus rosiglitazone baseline.

The absolute TEA-associated increment was smaller in untreated diabetic myocardium (+5 ms) than in untreated control myocardium (+20 ms). The higher final post-TEA ½RT in diabetic preparations (65 ms vs. 38 ms in controls) reflects the markedly prolonged diabetic baseline and must not be interpreted as a greater incremental response to TEA.

## Summary of electrophysiological findings

Electrophysiological analyses demonstrated altered ventricular membrane properties following diabetes induction, most clearly characterized by prolonged baseline ½RT. Claims regarding membrane depolarization or action-potential stability are limited to effects supported by the revised statistical model. Tetraethylammonium altered the measured electrophysiological parameters, with ½RT showing clear post-exposure increases. No cross-endpoint ranking of effect magnitude is asserted.

Rosiglitazone was associated with different descriptive pre-post TEA patterns, but whether it statistically modified TEA responsiveness is determined by the group × TEA interaction. It did not normalize the prolonged diabetic baseline ½RT.

## DISCUSSION

The present study examined the effect of broad TEA-sensitive potassium-conductance blockade on ventricular electrophysiological properties in streptozotocin-induced diabetic rat papillary muscle and assessed the influence of rosiglitazone. The main observations were: (i) diabetic myocardium had markedly prolonged baseline ½RT; (ii) TEA increased ½RT in all groups, but the absolute increment was smaller in untreated diabetic than untreated control myocardium; and (iii) rosiglitazone was associated with distinct descriptive response patterns that require confirmation by the group × TEA interaction. These results characterize an integrated tissue response to a non-selective blocker and do not identify a specific potassium current or channel subtype.

### Diabetes-induced ventricular electrical remodeling

Diabetic cardiomyopathy is increasingly recognized as a complex myocardial disorder that involves metabolic dysregulation, oxidative stress, mitochondrial dysfunction, fibrosis, inflammation, and electrophysiological remodeling. These factors contribute to impaired myocardial performance and increased arrhythmogenic susceptibility, independent of coronary artery disease or hypertension (^1^,^2^,^3^).

In this study, diabetic animals developed persistent hyperglycemia accompanied by impaired body weight gain and significant electrophysiological abnormalities. Diabetic papillary muscles exhibited depolarized RMPs and markedly prolonged repolarization compared with controls. These findings are consistent with previous studies demonstrating that chronic hyperglycemia causes substantial remodeling of cardiac ion channels and electrical signaling pathways (^6^,^13^).

Several mechanisms may contribute to diabetes-associated electrical remodeling. Chronic hyperglycemia promotes oxidative stress, advanced glycation end-product formation, mitochondrial dysfunction, and abnormalities in intracellular calcium handling, all of which may influence membrane excitability and ion-channel function (^2^,^17^,^18^). Previous studies have reported altered Kv4.2, Kv4.3, and Kir2.x expression in diabetic myocardium (^6^,^8^,^14^), but these channels were not measured here. The prolonged baseline ½RT is consistent with impaired repolarization at the tissue level; it does not establish the molecular basis of that abnormality.

### Functional importance of TEA-sensitive potassium conductances

Ventricular repolarization is maintained by the coordinated activity of multiple potassium currents, including Ito, IKr, IKs, and IK1. Together, these currents determine action potential duration and preserve repolarization reserve under physiological conditions (^7^,^12^).

TEA inhibits several potassium conductances in a concentration-dependent and non-selective manner. The observed increase in ½RT therefore demonstrates that the aggregate set of conductances sensitive to 2 mmol/L TEA contributes to ventricular voltage recovery. It does not permit attribution to Ito, IKr, IKs, IK1, Kv4.x, Kir2.x, or any other individual current or channel subtype.

Contrary to our original interpretation, the absolute TEA-associated prolongation was smaller in untreated diabetic myocardium (+5 ms) than in untreated controls (+20 ms), although the final ½RT remained much longer in diabetes (65 vs. 38 ms). The higher final value is principally explained by the markedly prolonged diabetic baseline (60 vs. 18 ms), not by a greater incremental response to TEA. A blunted increment may reflect a ceiling effect, reduced availability of TEA-sensitive conductance, altered action-potential morphology, or other diabetes-related changes; however, the present design cannot distinguish among these possibilities. Accordingly, we do not infer enhanced dependence on residual potassium-channel activity from these data.

Our findings also align with prior reports indicating that suppression of outward potassium currents not only prolongs ventricular repolarization but also increases susceptibility to afterdepolarizations and arrhythmias (^8^,^19^).

### Rosiglitazone and electrophysiological modulation

Rosiglitazone is a PPARγ agonist primarily used to improve insulin sensitivity and glycemic control. However, increasing evidence suggests that PPARγ signaling affects several pathways in cardiac electro-physiology, including oxidative stress regulation, mitochondrial function, calcium handling, and ion channel expression (^13^,^21^,^22^).

Rosiglitazone-treated preparations showed descriptive pre-post TEA patterns that differed from their untreated counterparts. Whether rosiglitazone statistically modified the TEA response must be established by the group × TEA interaction and adjusted contrasts; the present descriptive differences alone are insufficient to establish effect modification.

Any such effect would concern the integrated response to non-selective TEA-sensitive conductance blockade rather than a specific potassium channel.

Our findings are consistent with prior studies reporting direct electrophysiological effects of rosiglitazone on ventricular papillary muscles and cardiac ion channel activity (^20^,^23^). The underlying mechanisms remain incompletely understood but may involve modulation of potassium channel expression, cellular redox state, or mitochondrial bioenergetics.

Notably, rosiglitazone improved metabolic status and partially resolved diabetes-associated body weight loss and hyperglycemia. However, it did not fully normalize repolarization abnormalities, suggesting that glycemic control alone may not be sufficient to reverse established electrical remodeling in diabetic myocardium.

### Clinical implications

Impaired ventricular repolarization reserve is increasingly recognized as a critical substrate for ventricular arrhythmias and sudden cardiac death. Patients with DM exhibit a higher incidence of QT interval prolongation, ventricular tachyarrhythmias, and sudden cardiac death than non-diabetic individuals (^7^,^9^).

The present findings show marked baseline repolarization prolongation in diabetic myocardium and an altered response pattern after broad TEA-sensitive potassium-conductance blockade. Because the absolute TEA-associated increment was not enhanced in untreated diabetic tissue, the data do not support increased pharmacological sensitivity to TEA. The findings are compatible with diabetes-associated impairment of integrated repolarization, but the responsible ionic mechanisms remain unresolved.

From a translational perspective, preserving potassium channel function and repolarization reserve may offer a potential therapeutic strategy to reduce cardiovascular and arrhythmic complications in DM.

### Study limitations

Several limitations should be acknowledged. First, TEA is a relatively non-selective potassium-channel blocker, and 2 mmol/L TEA can inhibit multiple conductance components. Individual potassium currents were not measured by voltage clamp or patch clamp;

therefore, neither the affected current nor a specific channel subtype can be identified. The findings should be interpreted as a tissue-level response to aggregate TEA-sensitive potassium-conductance blockade.

Second, molecular expression of Kv4.2, Kv4.3, Kir2.x, or components underlying IKr and IKs was not assessed. Consequently, channel remodeling remains a literature-based hypothesis rather than a mechanism demonstrated by this study. Third, the pronounced baseline prolongation in diabetes may constrain the additional measurable increase after TEA (a possible ceiling effect), which limits direct comparison of raw change magnitudes.

Fourth, recordings were obtained at a single stimulation frequency, preventing evaluation of rate-dependent effects. Fifth, ½RT is a useful tissue-level index but is not equivalent to a direct measurement of individual ionic currents or clinical QT behavior. Finally, the STZ-induced rat model reproduces selected features of diabetic cardiomyopathy but cannot fully represent human disease.

### Future perspectives

Future studies integrating intracellular electrophysiology, patch-clamp recordings, optical mapping, calcium imaging, and molecular ion-channel profiling are warranted. Investigation of mitochondrial signaling pathways, oxidative stress mechanisms, and potassium channel remodeling may further clarify the mechanisms underlying impaired repolarization reserve in diabetic myocardium.

In addition, research on potassium channel-targeted therapies and novel metabolic interventions may provide promising strategies to reduce arrhythmogenic risk in diabetic cardiomyopathy.

In conclusion, streptozotocin-induced diabetes was associated with markedly prolonged baseline ventricular ½RT. Broad blockade with 2 mmol/L TEA further increased ½RT in all groups; however, the absolute increment was smaller in untreated diabetic than control myocardium (+5 vs. +20 ms). The higher post-TEA value in diabetes therefore reflects its prolonged baseline and should not be interpreted as greater TEA sensitivity or increased dependence on residual potassium-channel activity.

Rosiglitazone improved metabolic indices and was associated with different descriptive electro-physiological response patterns, but formal effect modification must be established by the group × TEA interaction. Rosiglitazone did not normalize diabetic repolarization.

Collectively, the findings support altered integrated ventricular repolarization in diabetes while remaining agnostic about the responsible channel subtype. Direct current measurements, selective blockers, channel-expression analyses, and complete interaction testing are required before a specific potassium-channel mechanism or arrhythmogenic consequence can be established.

## Data Availability

datasets related to this article will be available upon request to the corresponding author.

## References

[1] Jia G, Hill MA, Sowers JR (2018). Diabetic cardiomyopathy: an update of mechanisms contributing to
this clinical entity. Circ Res.

[2] Tan Y, Zhang Z, Zheng C, Wintergerst KA, Keller BB, Cai L (2020). Mechanisms of diabetic cardiomyopathy and potential therapeutic
strategies: preclinical and clinical evidence. Nat Rev Cardiol.

[3] Dillmann WH (2021). Diabetic cardiomyopathy. Circ Res.

[4] Nakamura M, Sadoshima J (2020). Mechanisms of physiological and pathological cardiac
hypertrophy. Nat Rev Cardiol.

[5] Riehle C, Abel ED (2020). Insulin signaling and heart failure. Circ Res.

[6] Liu X, Wang Y, Wang X, Zhang Y, Li H, Chen J (2021). Diabetes-associated cardiac ion channel remodeling and
arrhythmogenesis. Cardiovasc Diabetol.

[7] Varró A, Baczkó I (2021). Cardiac ventricular repolarization reserve: a principle for
understanding drug-related proarrhythmic risk. Br J Pharmacol.

[8] Nerbonne JM, Guo W (2022). Regulation of cardiac potassium channel expression and
function. Physiol Rev.

[9] Tomaselli GF, Zipes DP, Libby P, Bonow RO, Mann DL, Tomaselli GF, Bhatt DL, Solomon SD, Braunwald E (2024). Braunwald's Heart Disease.

[10] Yap YG, Camm AJ (2021). Drug-induced QT prolongation and torsades de
pointes. Heart.

[11] Kowluru RA, Chan PS (2022). Oxidative stress and diabetic cardiomyopathy. Free Radic Biol Med.

[12] Chiamvimonvat N, Chen-Izu Y, Clancy CE, Deschenes I, Dobrev D, Heijman J (2017). Potassium currents in the heart: functional roles in
repolarization, arrhythmia and therapeutics. J Physiol.

[13] Wang Y, Zhang Y, Li X, Chen H (2022). PPARγ signaling and cardiac electrophysiological
remodeling in diabetes mellitus. Front Cardiovasc Med.

[14] Shao Y, Li H, Wang J, Zhang X, Chen Y (2023). Potassium channel remodeling in diabetic cardiomyopathy:
mechanisms and therapeutic implications. Front Physiol.

[15] Bugger H (2021). Mitochondrial mechanisms in diabetic
cardiomyopathy. Rev Endocr Metab Disord.

[16] Bertero E, Maack C (2018). Metabolic remodelling in heart failure. Nat Rev Cardiol.

[17] Brown DI, Griendling KK (2021). Regulation of signal transduction by reactive oxygen species in
the cardiovascular system. Circ Res.

[18] Murphy E, Steenbergen C (2021). Mechanisms underlying acute protection from cardiac
ischemia-reperfusion injury. Physiol Rev.

[19] Ravens U, Cerbai E (2020). Role of potassium currents in cardiac electrophysiology and
arrhythmogenesis. Pharmacol Ther.

[20] Choisy SCM, Arberry LA, Hancox JC, James AF (2004). Acute effects of rosiglitazone on cardiac electrophysiological
properties and ion channels. Br J Pharmacol.

[21] Soccio RE, Chen ER, Lazar MA (2014). Thiazolidinediones and the promise of insulin sensitization in
type 2 diabetes. Cell Metab.

[22] He X, Zhao Y, Wang L, Zhang M (2023). PPARγ signaling in cardiovascular disease and metabolic
regulation. Int J Mol Sci.

[23] Kavak S, Emre M, Tetiker T, Kavak T, Kolcu Z, Günay ? (2008). Effects of rosiglitazone on altered electrical left ventricular
papillary muscle activities of diabetic rat. Naunyn Schmiedeberg's Arch Pharmacol.

[24] Perciedu Sert N, Ahluwalia A, Alam S, Avey MT, Baker M, Browne WJ (2020). Reporting animal research: explanation and elaboration for the
ARRIVE guidelines 2.0. PLoS Biol.

[25] National Research Council (2011). Guide for the Care and Use of Laboratory Animals.

[26] Faul F, Erdfelder E, Buchner A, Lang AG (2009). Statistical power analyses using G*power 3.1: tests for
correlation and regression analyses. Behav Res Methods.

